# Population‐specific effects of developmental temperature on body condition and jumping performance of a widespread European frog

**DOI:** 10.1002/ece3.2113

**Published:** 2016-04-03

**Authors:** Sanja Drakulić, Heike Feldhaar, Duje Lisičić, Mia Mioč, Ivan Cizelj, Michael Seiler, Theresa Spatz, Mark‐Oliver Rödel

**Affiliations:** ^1^ Museum für NaturkundeLeibniz Institute for Evolution and Biodiversity ScienceBerlinGermany; ^2^ Department of Animal Ecology IBayreuth Centre of Ecology and Environmental Research (BayCEER)University of BayreuthBayreuthGermany; ^3^ Department of Animal PhysiologyFaculty of ScienceUniversity of ZagrebZagrebCroatia; ^4^ Zoological Garden of ZagrebZagrebCroatia; ^5^ Department of Animal Ecology and Tropical BiologyUniversity of WürzburgWürzburgGermany

**Keywords:** Amphibians, ectotherms, physiological traits, plasticity, thermal adaptation

## Abstract

All physiological processes of ectotherms depend on environmental temperature. Thus, adaptation of physiological mechanisms to the thermal environments is important for achieving optimal performance and fitness. The European Common Frog, *Rana temporaria*, is widely distributed across different thermal habitats. This makes it an exceptional model for studying the adaptations to different thermal conditions. We raised tadpoles from Germany and Croatia at two constant temperature treatments (15°C, 20°C), and under natural temperature fluctuations (in outdoor treatments), and tested how different developmental temperatures affected developmental traits, that is, length of larval development, morphometrics, and body condition, as well as jumping performance of metamorphs. Our results revealed population‐specific differences in developmental time, body condition, and jumping performance. Croatian frogs developed faster in all treatments, were heavier, in better body condition, and had longer hind limbs and better jumping abilities than German metamorphs. The populations further differed in thermal sensitivity of jumping performance. While metamorphs from Croatia increased their jumping performance with higher temperatures, German metamorphs reached their performance maximum at lower temperatures. These population‐specific differences in common environments indicate local genetic adaptation, with southern populations being better adapted to higher temperatures than those from north of the Alps.

## Introduction

Ectotherms are unable to generate a significant amount of metabolic body heat (Hillman et al. 2009). Consequentially, all physiological processes and thus the performance of ectotherms strongly depended on environmental temperatures (Angilletta 2009). The capacity of behavioral thermoregulation might be constrained by the unavailability of favorable microhabitats or trade‐offs between energy needs and available activity periods (Huey and Slatkin 1976). Therefore, adaptation to local thermal conditions is of great importance to achieve optimal performance and thus fitness (Kingsolver and Huey 2003; Angilletta 2009; Keller and Seehausen 2012). Such adaptations may be genetically fixed or phenotypically plastic. Selection‐driven genetic adaptation may lead to locally specialized phenotypes (Kawecki and Ebert 2004). Phenotypic plasticity, in contrast, enables single genotypes to produce multiple phenotypes in different environments, allowing the expression of a broader range of morphological, behavioral, or physiological characters and, therefore, survival under a wide set of environmental conditions (West‐Eberhard 2003).

Metabolic rates, morphology, and locomotor performance can directly or indirectly influence performance and thus fitness of ectotherms (Arnold 1983; Angilletta et al. 2002; Kingsolver and Huey 2003). They are therefore commonly used to assess the influence of environmental temperatures (Carey 2005; Seebacher and Franklin 2012). For example, development under lower temperatures usually leads to a longer developmental period, but bigger body size (Atkinson 1994, 1995). Body size, in turn, affects survival, locomotor performance, and reproductive success (Zug 1972; Wilbur and Collins 1973; Schmidt‐Nielsen 1975; Blueweiss et al. 1978; Wikelski and Romero 2003), while locomotion impacts success in foraging (Putnam and Bennett 1981) or antipredator behavior (Wassersug and Sperry 1977). Therefore, temperature can be a strong selective factor, and testing how physiological traits, morphology, and locomotor performance respond to different thermal environments can provide valuable insight into local adaptations (Angilletta et al. 2002, 2004).

**Figure 1:**
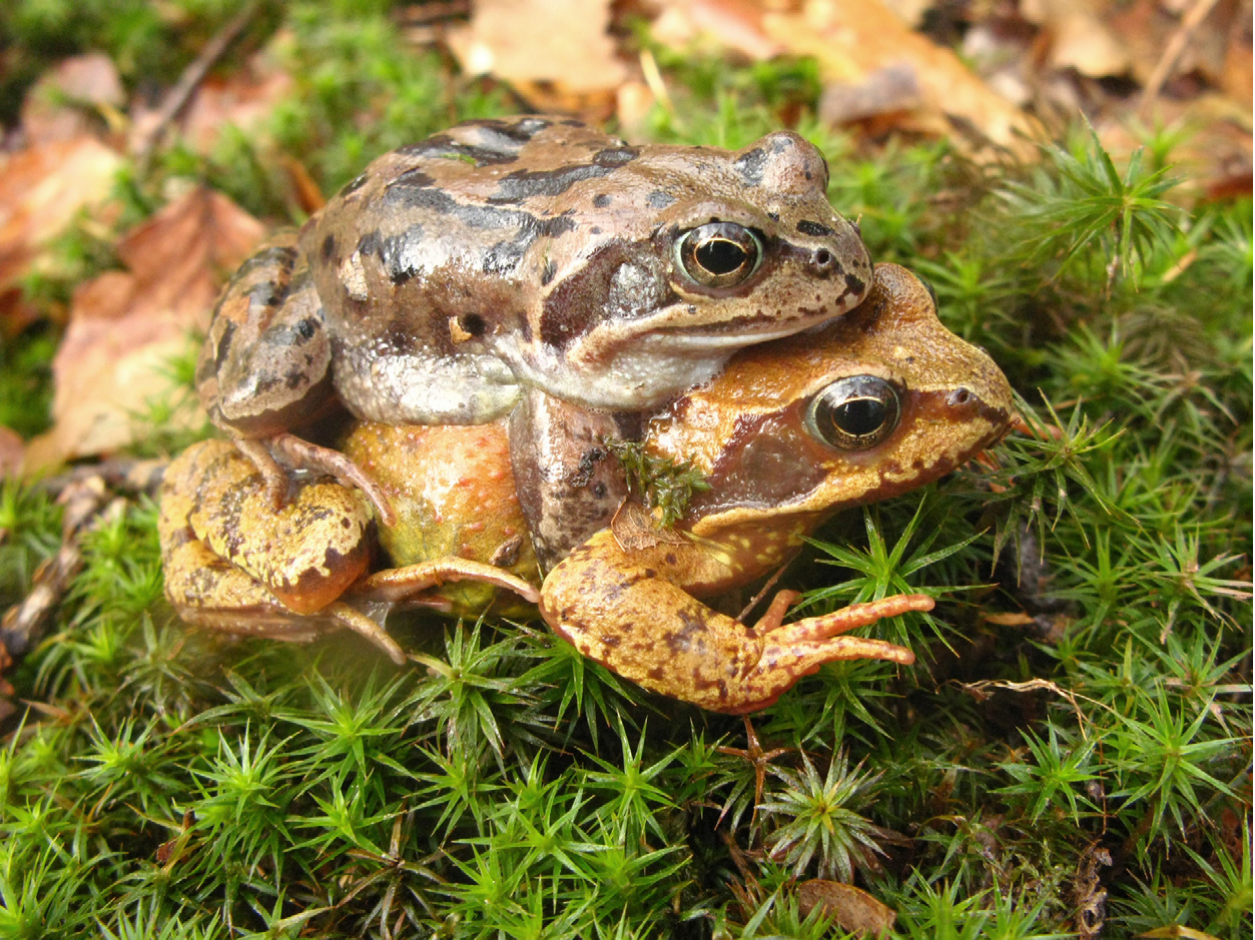
*Rana temporaria* (European Common Frog); Steigerwald, Bavaria, Germany.

Amphibians are particularly sensitive to thermal and hygric conditions (Hillman et al. 2009; Blaustein et al. 2010), which makes them excellent models to test the differences in local thermal adaptation. The European Common Frog, *Rana temporaria* Linnaeus, 1758 (Fig. 1), is widely distributed in open and forested habitats, ranging from northern Spain to western Siberia, and from northern Scandinavia to northern Greece (Gollmann et al. 2014). Its persistence under a wide range of environmental conditions makes it ideal for studying population‐specific physiological adaptations to local thermal environments. The species' ability to respond to different environmental conditions and ecological constraints, such as desiccation risk or growth season duration, was demonstrated, for example, concerning growth and developmental rates, as well as morphology (Merilä et al. 2000; Laurila et al. 2002; Laugen et al. 2003a,b; Lind and Johansson 2007; Lind et al. 2008). Local adaptation was evident even in the presence of high gene flow at some populations, indicating these traits are indeed under strong selection pressure (Lind et al. 2010; Richter‐Boix et al. 2010; Muir et al. 2014). The majority of the studies so far focused on the northern range of the species, that is, Scandinavia and Great Britain, neglecting southern populations (e.g., Merilä et al. 2000; Laugen et al. 2003b; Lind and Johansson 2007; Muir et al. 2014).

We therefore herein aimed to seek the evidence of adaptation to local thermal conditions of populations from the southern and central part of the distribution range. We raised tadpoles from Germany (north of the Alps: colder) and Croatia (south of the Alps: warmer) under two constant temperature regimes (15° and 20°C) and under respective natural fluctuating temperatures, to test for population‐ and temperature‐dependant differences in length of larval period, morphometric traits, and jumping performance. We predicted that tadpoles and metamorphs would perform best under their original thermal conditions and exhibit population‐specific thermal adaptations. In particular, when developed under constantly increased temperatures, froglets from the south should perform better (e.g., display better body condition and/or increased jumping abilities) than froglets from the north.

## Materials and Methods

### Study sites

*Rana temporaria* uses temporary and permanent forest ponds as breeding sites at both study regions. On 11 April 2013, we collected newly deposited *R. temporaria* eggs from 10 clutches at a forest pond (FS06) in the Steigerwald, northern Bavaria, Germany (49°55′N, 10°33′E, 409 m asl; see Grözinger et al. 2014), hereafter referred to as GER. Experiments were performed from April to July 2013 in the ecological field station in Fabrikschleichach. On 16 March 2014, we collected newly deposited eggs of 10 clutches from a forest pond (RJ01) in Medvednica (45°53′N, 16°00′E, 400 m asl), close to Zagreb, Croatia, hereafter referred to as CRO. Experiments were conducted from March to June 2014 at the Zagreb Zoo. Even though both study areas are within the temperate zone, their climate differs. Croatia's lowland climate is generally warmer, with hot, dry summers, and cold winters. The average annual temperature in CRO is 10.8°C (Croatian Meteorological and Hydrological Service; Maksimir weather station, 45°49′N, 16°02′E). In January, the coldest month of the year, average temperature is 0°C. With an average temperature of 20.9°C, July is the warmest month. In GER, the average annual temperature is 8.2°C, with an average of −0.6°C in January and 17.4°C in July (Deutscher Wetterdienst; Ebrach weather station, 49°51′N, 10°30′E).

### Experimental procedures

#### Climate chambers and outdoor treatments

The eggs of different clutches were kept in separate containers at a constant temperature of 7–8°C for 5 days. This mirrored the water temperature of both original ponds during egg collection. From each clutch, we assigned eggs to three developmental treatments. Two climate chamber treatments had constant developmental temperatures of 15°C (low temperature treatment, T15) or 20°C (high temperature treatment, T20). These temperatures were within the ranges we measured in ponds of both study sites. The third treatment was an outdoor treatment (OT), designed to mimic the natural conditions. Here, temperature fluctuated in accordance with local, ambient temperatures (Fig. S1). In the following, we label a particular developmental treatment of a particular population using treatment population abbreviation, for example, T15‐GER for eggs/tadpoles raised at 15°, originating from Steigerwald, Germany.

Tadpoles hatched almost simultaneously across the 10 clutches within each treatment. After reaching developmental stage 25 (Gosner 1960: free swimming and feeding), we randomly selected 12 tadpoles per clutch for each of the climate chamber treatments and placed them by six into a plastic container filled with 1.2 L of mixed deionized/spring water (pH = 6.5–7.0, conductivity = 150–200 *μ*S/cm). Light conditions in the climate chambers were 16‐L:8‐D h. For OT, 10 tadpoles per clutch were placed into a container with 5 L of the original pond water (GER: pH = 6.9, conductivity = 110 *μ*S/cm, CRO: pH = 7.0, conductivity = 320 *μ*S/cm). The bottoms of the OT containers were covered with a 2 cm layer of soil and leaf litter. They were placed into shade outside and protected from predators by plastic gauze coverage. For each population, we tested a total of 120 tadpoles (6 tadpoles × 20 containers) per climate chamber treatment and 100 tadpoles (10 tadpoles × 10 containers) in the respective outdoor treatment. All tadpoles not used in the experiments were instantly released at their original ponds.

The tadpoles were fed *ad libitum* with commercial fish food (TetraTabiMin^®^; Tetra, Melle, Germany). In climate chamber treatments, water was exchanged 2–3 times per week. At the outdoor treatments, water was only changed when water chemistry exceeded predefined ranges (pH out of 6.2–7.5, or CD >370 *μ*S/cm; AL15MultiMeter Instrument; AquaLytic^®^, Dortmund, Germany). We recorded water temperature every 3 h in each OT container with a thermologger (Theromochron^®^ iButtons© DS1922 l, ±0.5°C; Embedded Data Systems, Lawrenceburg, KY). Temperatures are expressed as means of 10 containers for each time point.

#### Developmental time

Developmental period was determined from egg collection until peak of metamorphosis (Gosner stage 42: emergence of front limbs; Gosner 1960). Tadpoles from the same matriline were not independent samples. Thus, the matriline and not a tadpole is a sample unit, and the duration of development is expressed as a mean value of all tadpoles from one clutch (*n* = 10 clutches per treatment). Tadpoles were kept until metamorphosis was completed (Gosner stage 45: tail totally reabsorbed). Then, we randomly chose 30 froglets from each treatment and population, for morphometric measurements and jumping performance tests.

#### Morphometric traits and body condition

We measured the snout‐vent (SVL) and length of hind legs, from hip to toe (LL) of metamorphs (Gosner stage 45; calliper accuracy ± 0.5 mm), and weight (PS‐200HTP scale, Voltcraft^®^, accuracy ± 0.06 g; Conrad Electronics, Hirschau, Germany). Again, to correct for potential matriline effect, we expressed the morphometric values (SVL, mass, LL) as mean values for each clutch. To test for allometric differences among metamorphs from different treatments, we calculated relative leg length (leg length index; LLI), using the equation (1) (Loman 2002; Choi et al. 2003):

(1) $$\text{Leg length index (LLI)}=\frac{\text{LL}}{\text{SVL}}$$

Body condition is an important trait, because energy reserves (body fat and/or proteins) may strongly influence survival and fitness (Reading 2007). Here, we use the scaled mass index (SMI) as a measure of body condition, because it is comparable among different populations or species (Peig and Green 2009; MacCracken and Stebbings 2012). We calculated SMI, using the equation (2), by Peig and Green (2009):

(2) $$\text{Scaled mass index (SMI)}=M_{i}{\left[\frac{L_{0}}{L_{i}}\right]}^{b_{\mathrm{SMA}}}$$

where *M*_i_ and *L*_i_ are measurements of mass and length of an individual, and *L*_0_ is an arbitrary value of *L* (e.g., the mean value within population(s)). In our study, *M*_i_ and *L*_i_ are expressed as a mean value of clutch members, within a developmental treatment. As a reference for size measurement (*L*_0_), we selected the mean SVL of metamorphs from the outdoor treatments of both populati‐ons (*L*_0_ = 18.8 mm; *n*(GER) = 93, *n*(CRO) = 86, total = 179). The scaling exponent, *b*_SMA_, represents a species‐specific value of the relation between size and body mass (Peig and Green 2010). It was estimated by the standardized major axis (SMA) regression of ln‐transformed values of mass on body length (*lmodel2* package in R; Legendre 2014; R Development Core Team 2014). To include a representative range of size measurements for the *b*_SMA_ (Peig and Green 2010), we used data of outdoor treatment metamorphs, and adults measured during field surveys of our study sites (*b*_SMA_ = 3.08; *n*(adults) = 522, *n*(froglets) = 179, total = 701).

#### Jumping performance

Jumping performance of froglets was tested under three experimental temperatures: 15°C, 20°C, and 25°C (hereafter E15, E20, and E25). These temperatures fall into the range of environmental temperatures at both study sites during metamorphosis. Prior to testing, froglets (*n* = 30 per treatment) were acclimatized in a thermal chamber for a minimum of 1.5 h. We heated or cooled the animals by not more than 5°C at once. If, for example, E15 followed E25, they spent two additional hours at the median temperature, E20. Jumping was tested within a 100 × 50 cm arena. Froglets were ventrally marked with neutral edible dye (Orizaola and Laurila 2009) and stimulated to jump by gently touching the urostyle. Landing points were indicated on the paper by color marks. After three jumps, we measured the distance between the marks (end to end of hind legs). The longest jump was taken for the analyses, and the frog was cleaned with spring water.

Jumping distance is positively affected by body size and hind leg length (Zug 1978; Tejedo et al. 2000; Herrel et al. 2012). To correct the jumping distance for size, we analyzed the relations of morphometric traits and jumping distance. Among all morphometric traits, LLI showed the highest correlation with max. jumping distance (see Appendix: Table S1). Therefore, we adjusted maximal jumping distance for LLI, by calculating the max. jump index (MJI) as in equation (3):

(3) $$\text{Max. jump index (MJI)}=\frac{\text{Max. jumping distance}}{\text{LLI}}$$

### Statistical analysis

We tested all data for normality of distribution using Shapiro–Wilk test. Developmental temperature data of the outdoor treatments and developmental time data were not normally distributed, and thus, we compared them with Kruskal–Wallis rank‐sum tests.

We compared the morphometric traits (SVL, mass, SMI, LL, and LLI) of animals within a population (GER or CRO), but raised under different treatments. Then, we compared the animals from the same treatment, but of different origin (GER vs. CRO). Normally distributed data (mass and SMI) were compared using one‐way ANOVA with Tukey post hoc test (intrapopulation analysis) and Welch two sample *t*‐test (interpopulation analysis). Non‐normally distributed data (SVL, LL, and LLI) were tested using Kruskal–Wallis rank‐sum test and post hoc pairwise Wilcoxon rank‐sum test, with the FDR method (Benjamini and Hochberg 1995) of *P*‐value correction for multiple comparisons.

To analyze the overall influence of developmental and experimental temperatures on jumping performance, we constructed linear mixed‐effect models, separated by populations. We treated MJI as a dependent variable, treatment, and experimental temperature as fixed, and clutch as a random factor. The models were constructed and analyzed using *nlme* (Pinheiro et al. 2015) and *lme4* (Bates et al. 2015) packages in R (R Development Core Team 2014). Due to high collinearity (Zuur et al. 2010), it was not possible to include some significant factors (population, treatment, and experimental temperature) in the model and thus examine their interactions. Therefore, we used one‐way repeated‐measures ANOVA with post hoc pairwise *t*‐test (paired) and Welch two sample *t*‐test for examining the effect of these factors on jumping performance. We compared jumping performance of froglets from a single developmental treatment, jumping at different experimental temperatures (e.g., T15‐GER jumping at E15, E20 and E25; one‐way repeated‐measures ANOVA and post hoc pairwise comparisons using paired *t*‐test, with FDR *P*‐value correction). Repeated measurements data sets were tested for sphericity using Mauchly's test (*ez* package in R; Lawrence 2015), which indicated violation of sphericity at OT‐CRO. Therefore, we corrected the degrees of freedom for OT‐CRO using Huynh–Feldt estimate of sphericity (*ɛ *= 0.85). To test for population influence on jumping performance, we compared froglets from the same treatment, jumping at the same experimental temperature, but originating from different populations (e.g., T15‐GER vs. T15‐CRO, jumping at E15, E20 and finally E25), with Welch two sample *t*‐tests. We performed all calculations and analysis using R (R Development Core Team 2014, visualizations: *ggplot2*; Wickham 2009).

### Ethics statement

We declare that animals were cared for in accordance with guidelines on the animal care and use compiled by the American Society of Ichthyologists and Herpetologists (ASIH), The Herpetologists' League (HL) and the Society for the Study of Amphibians and Reptiles (SSAR). Due to the German Protection of Animal Act (“Tierschutzgesetz,” http://www.gesetze-im-internet.de/tierschg/BJNR012770972.html; §1/§7; 9 December 2010; last accessed on 13 August 2015) and Croatian Regulative on the protection of animals used for scientific purposes (http://narodne-novine.nn.hr/clanci/sluzbeni/2013_05_55_1129.html; §1/§6f; 08 May 2013; last accessed on 13 August 2015), painless experiments and observations of vertebrates neither require permission nor disclosure. The vertebrates involved, *R. temporaria* tadpoles and metamorphs, experienced no pain, suffering, complaints, or harm. Thus, no Institutional Animal Care and Use Committee (IACUC) or ethics committee approved this study, as this was not required by German or Croatian law. In Germany, Higher Nature Conservation Authority of Lower Franconia – “Regierung von Unterfranken” approved the research in accordance with the Federal Conservation of Nature and Landscape Act (“Bundesnaturschutzgesetz”). In Croatia, *R. temporaria* is not protected by the law, so no sampling permits were necessary (Ministry of Environmental and Nature Protection, decision class: UP/I‐612‐07/13‐48/107, permit nr. 515‐07‐1‐1‐1‐14‐6; Zagreb, 28 January 2014).

## Results

### Developmental time

**Figure 2:**
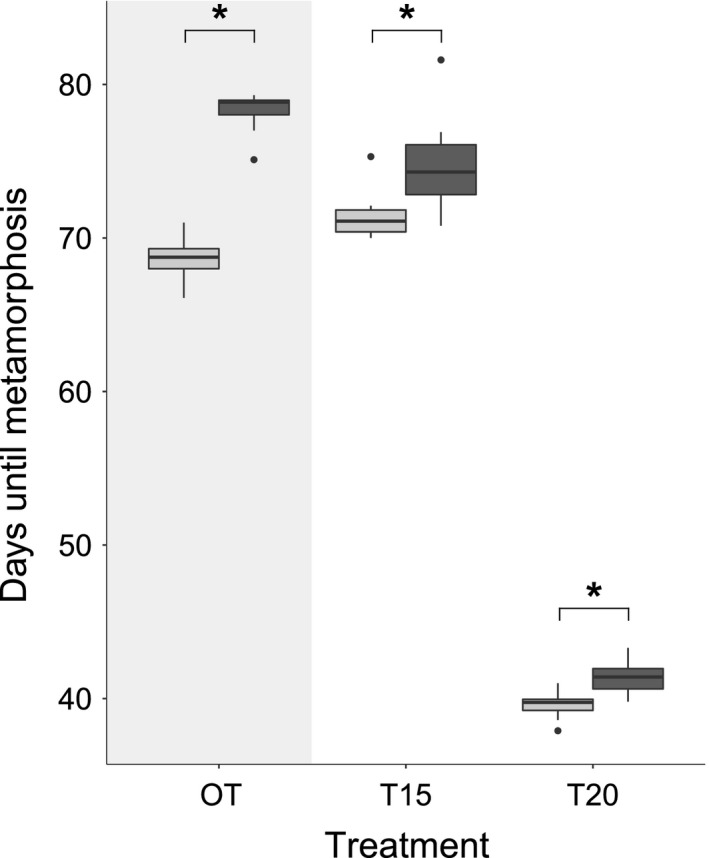
Developmental time of *Rana temporaria* tadpoles developing under different temperature regimes. Developmental time was measured in days from egg collection to metamorphosis (Gosner stage 42). Tadpoles originated from Germany (dark gray; GER) and Croatia (light gray; CRO) and developed either outdoor under natural temperature fluctuations (OT – outdoor treatment, shaded), or in climate chambers at constant temperatures of 15°C (T15) or 20°C (T20). Significant differences between populations GER and CRO are indicated with an asterisk. Sample size for each population and treatment was *n* = 10.

Survival rates were high in all treatments and populations (GER – OT: 93%, T15: 94.2%, T20: 95.8%; CRO – OT: 95%, T15: 85%, T20: 90%). Developmental temperatures of the outdoor treatments (time from egg collection to last metamorph) differed significantly among CRO and GER (Kruskal–Wallis test, *χ* ^2^ = 81.58, *df *= 1, *P* < 0.0001; *n*(GER) = 640, *n*(CRO) = 569). Mean water temperature in GER was lower (12.4°C; range: 4.7–26.0°C, SD = 3.8), compared to CRO (14.1°C, range: 7.5–23.4°C, SD = 3.4; for daily temperature fluctuations see Fig. S1). Tadpoles from OT‐CRO developed at higher temperatures and metamorphosed significantly faster than OT‐GER (median nr. of days – OT: CRO = 68.8, GER = 78.9; Kruskal–Wallis test, *χ* ^2^ = 14.31, *df *= 1, *P* = 0.0002; Fig. 2).

In constant temperature treatments, tadpoles from both populations developed slower at lower (T15) compared to higher temperature (T20; Fig. 2). When comparing populations, tadpoles from CRO developed significantly faster in both constant temperature treatments (median nr. of days – T15: CRO = 71.1, GER = 74.3, T20: CRO = 39.8, GER = 41.4; Kruskal–Wallis test, T15: *χ* ^2^ = 8.04, *df *= 1, *P* = 0.005; T20: *χ* ^2^ = 9.89, *df *= 1, *P* = 0.002; Fig. 2).

### Morphometric traits and body condition

#### Outdoor treatment

**Figure 3:**
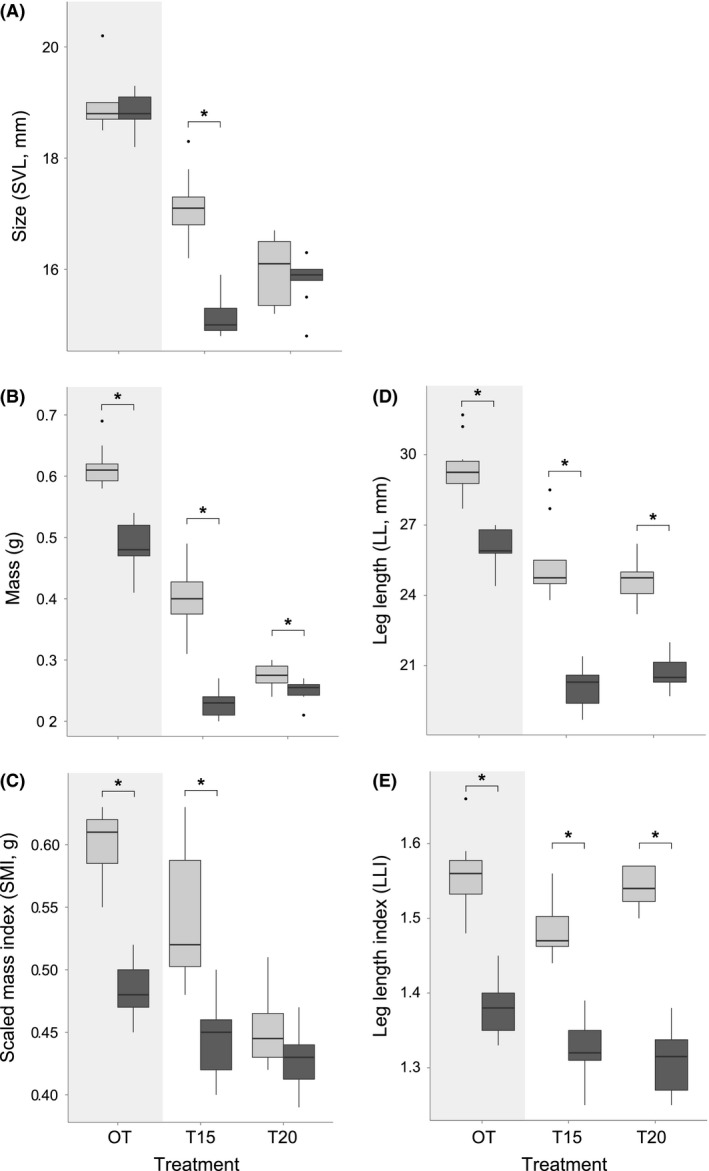
Morphometric traits and body condition of *Rana temporaria* froglets (Gosner stage 45) developing under different temperature regimes. Tadpoles originated from Germany (dark gray; GER) and Croatia (light gray; CRO) and developed under three temperature regimes (OT, T15, T20 – see text for details). After reaching Gosner stage 45 (total tail reabsorption), size (A; SVL, mm) and mass (B; g) were measured, and scaled mass index (C; SMI, g) was calculated (see text). Also, leg length (D; LL, mm, from hip to toe) was measured, and leg length index (E; LLI) calculated (LLI = LL/SVL). Significant differences between populations GER and CRO are indicated with an asterisk. Sample size for OT‐GER and T15‐GER was *n* = 9; for all other treatments was *n* = 10.

When compared to constant temperature treatments, animals from OT of both GER and CRO were bigger, heavier, in better body condition and had absolutely longer hind limbs (Fig. 3, Table 1). Metamorphs from OT‐CRO were of the same size, but heavier, in better body condition, and had absolutely and relatively longer hind limbs, than OT‐GER (Fig. 3, Table 2).

**Table 1:** Comparisons of morphometric traits and body condition of *Rana temporaria* froglets (Gosner stage 45) from different developmental treatments of the same population. Tadpoles originated from (A) Germany (GER) and (B) Croatia (CRO) and developed under three temperature regimes (OT, T15, T20 – see text for details). We used Kruskal–Wallis test (*χ* ^2^(*df*), *P*‐values) for size (SVL), hind leg length (LL), and leg length index (LLI) comparisons, with *P*‐values after Wilcoxon rank‐sum post hoc test (FDR *P*‐value adjustment method); and one‐way ANOVA (F(*df*)‐values, *P*‐values), for comparisons of mass and scaled mass index (SMI), with *P*‐values after Tukey post hoc test. Sample size was *n* = 9 for OT‐GER and T15‐GER; *n* = 10 for all other treatments

	SVL	LL	LLI		Mass	SMI
(A) GER						
Kruskal–Wallis test				One‐way ANOVA		
***χ*** ^**2**^ **(2)**	21.11	18.77	9.07	**F (2,25)**	202.8	10.49
*P*‐value	**<0.0001**	**<0.0001**	**0.01**	*P*‐value	**<0.0001**	**0.0005**
Pairwise Wilcoxon rank‐sum post hoc test				Tukey post hoc test		
***P*** **(OT‐T15)**	**0.0006**	**0.0006**	**0.03**	***P*** **(OT‐T15)**	**<0.0001**	**0.02**
***P*** **(OT‐T20)**	**0.0006**	**0.0006**	**0.02**	***P*** **(OT‐T20)**	**<0.0001**	**0.0004**
***P*** **(T15‐T20)**	**0.008**	0.15	0.71	***P*** **(T15‐T20)**	0.31	0.36
(B) CRO						
Kruskal–Wallis test				One‐way ANOVA		
***χ*** ^**2**^ **(2)**	23.78	18.83	11.16	**F (2, 27)**	210.6	37.35
*P*‐value	**<0.0001**	**<0.0001**	**0.004**	*P*‐value	**<0.0001**	**<0.0001**
Pairwise Wilcoxon rank‐sum post hoc test				Tukey post hoc test		
***P*** **(OT‐T15)**	**0.0003**	**0.0006**	**0.008**	***P*** **(OT‐T15)**	**<0.0001**	**0.006**
***P*** **(OT‐T20)**	**0.0003**	**0.0005**	0.44	***P*** **(OT‐T20)**	**<0.0001**	**<0.0001**
***P*** **(T15‐T20)**	**0.002**	0.43	**0.008**	***P*** **(T15‐T20)**	**<0.0001**	**<0.0001**

Statistically significant differences (*P*‐values <0.05) are shown in bold.

**Table 2:** Comparisons of morphometric traits and body condition of *Rana temporaria* froglets (Gosner stage 45) from different populations. Tadpoles originated from Germany (GER) and Croatia (CRO) and developed under three temperature regimes (OT, T15, T20). We used Kruskal–Wallis test (*χ* ^2^(*df*), *P*‐values) for comparisons of SVL, LL, LLI; and Welch two sample *t*‐test (*t*‐values, *df*,* P*‐values) for comparisons of mass and SMI. Sample size was *n* = 9 for OT‐GER and T15‐GER; *n* = 10 for all other treatments

	SVL		LL		LLI		Mass			SMI		
	Kruskal–Wallis test						Welch two sample *t*‐test					
	*χ* ^2^ (1)	*P*‐value	*χ* ^2^ (1)	*P*‐value	*χ* ^2^ (1)	*P*‐value	*t*‐value	*df*	*P*‐value	*t*‐value	*df*	*P*‐value
OT‐GER – OT‐CRO	0.03	0.87	13.51	**0.0002**	13.52	**0.0002**	6.97	14.91	**<0.0001**	10.59	17.00	**<0.0001**
T15‐GER – T15‐CRO	13.56	**0.0002**	13.56	**0.0002**	13.61	**0.0002**	9.63	12.80	**<0.0001**	4.67	15.58	**0.0003**
T20‐GER – T20‐CRO	0.64	0.42	14.36	**0.0002**	14.47	**0.0001**	2.74	17.87	**0.01**	1.96	15.99	0.07

Statistically significant differences (*P*‐values <0.05) are shown in bold.

#### Climate chamber treatments

Comparing metamorphs of the same population, which developed in different treatments, we found significant differences in SVL between T15 and T20, in both GER and CRO (Fig. 3A, Table 1). While froglets from T15‐GER, despite having a longer developmental period, were significantly smaller than T20‐GER, froglets from T15‐CRO were significantly bigger than T20‐CRO. We found no difference in mass or SMI between T15‐GER and T20‐GER, while mass and SMI of T15‐CRO were significantly bigger than T20‐CRO (Fig. 3B and C, Table 1).

Comparing metamorphs from different populations, T15‐CRO froglets had significantly higher values in all traits – size, mass, and body condition, compared to T15‐GER. At T20, we found significant differences in mass, with T20‐CRO being heavier than T20‐GER, but not in SVL or SMI. SMI of T20‐CRO did show the same tendency as mass, but without statistical support (Fig. 3A–C, Table 2). Regardless of treatment, legs of froglets from CRO were significantly longer than those from GER, in absolute size and relative to SVL (Fig. 3D and E, Table 2).

### Jumping performance

**Figure 4:**
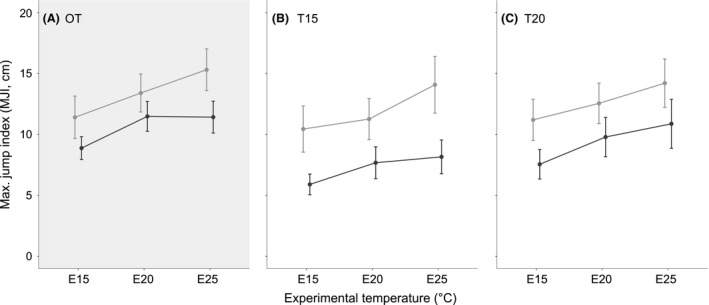
Jumping performance of *Rana temporaria* froglets (Gosner stage 45) developing under different temperature regimes. Tadpoles originated from Germany (dark gray; GER) and Croatia (light gray; CRO) and developed under three temperature regimes (A. OT, B. T15 and C. T20 – see text for details). After reaching Gosner stage 45, we tested jumping performance under three different experimental temperatures – 15°C (E15), 20°C (E20) and 25°C (E25) and calculated adjusted maximal jumping distance (max. jump index; MJI = max. jump/LLI, cm). Given are mean values ± SD. Sample size was *n* = 29 for OT‐CRO, and *n* = 30 for all other treatments.

Froglets from the outdoor treatments jumped further (max. jump. index, MJI) in comparison with constant temperature treatments, in both populations. Froglets from T15, generally jumped shorter distances compared to T20, in both populations (for the parameters and results of the linear mixed‐effect models, see Table S2). The overall correlation of experimental temperatures (E15, E20, and E25) with performance (MJI) was positive, in both populations, indicating that animals jumped further at higher experimental temperatures. However, in GER, this was not significant for all developmental treatments (see below; Fig. 4).

To test whether the two populations differ in thermal sensitivity of jumping performance, we compared frogs from the same developmental treatment (of GER or CRO), but jumping at different experimental temperatures (E15, E20, and E25). As visible in the performance curves shape (Fig. 4), froglets from CRO, regardless of developmental treatment, jumped significantly further with increasing temperature (Fig. 4, Table 3B). In GER, froglets reared at lower temperatures (OT and T15) improved performance at E20 (compared to E15), but in contrast to CRO, there was no further improvement at E25. In OT‐GER, there was no significant difference in MJI between E20 and E25, while froglets from T15‐GER showed a small, but significant increase. Only froglets from T20‐GER demonstrated a strong increase and jumped significantly further at E25, compared to E20 (Fig. 4, Table 3A).

**Table 3:** Comparisons of jumping performance of *Rana temporaria* froglets (Gosner stage 45) jumping under different experimental temperatures. Tadpoles originated from (A) Germany (GER) and (B) Croatia (CRO) and developed under three temperature regimes (OT, T15, T20). We compared adjusted maximal jumping distance (max. jump index, MJI = max. jump/LLI, cm) of froglets from the same developmental treatment, jumping at different experimental temperatures (E15, E20, E25), using one‐way repeated‐measures ANOVA and post hoc pairwise comparisons using paired *t*‐test with FDR *P*‐value correction method. Sample size was *n* = 29 for OT‐CRO, and *n* = 30 for all other treatments

	OT		T15		T20	
(A) GER						
One‐way repeated‐measures ANOVA						
	**F (2,58)**	***P*** **‐value**	**F(2,58)**	***P*** **‐value**	**F(2,58)**	***P*** **‐value**
	72.03	**<0.0001**	59.51	**<0.0001**	78.10	**<0.0001**
Pairwise paired *t*‐test						
	***P*** **‐value**		***P*** **‐value**		***P*** **‐value**	
E15‐E20	**<0.0001**		**<0.0001**		**<0.0001**	
E15‐E25	**<0.0001**		**<0.0001**		**<0.0001**	
E20‐E25	0.82		**0.04**		**0.0008**	
(B) CRO						
One‐way repeated‐measures ANOVA						
	**F (1.7, 47.6)** a	***P*** **‐value**	**F(2,58)**	***P*** **‐value**	**F(2,58)**	***P*** **‐value**
	76.28	**<0.0001**	54.11	**<0.0001**	53.33	**<0.0001**
Pairwise paired *t*‐test						
	***P*** **‐value**		***P*** **‐value**		***P*** **‐value**	
E15‐E20	**<0.0001**		**0.02**		**0.0001**	
E15‐E25	**<0.0001**		**<0.0001**		**<0.0001**	
E20‐E25	**<0.0001**		**<0.0001**		**<0.0001**	

a *df* adjusted using Huynh–Feldt estimate of sphericity (*ɛ *= 0.85).

Statistically significant differences (*P*‐values <0.05) are shown in bold.

To test for population differences in jumping performance, we compared froglets from CRO and GER raised at the same developmental treatment, and jumping at the same experimental temperature. Comparisons of MJI revealed that froglets from CRO jumped significantly further than froglets from GER under all experimental temperatures, and regardless of treatment (Fig. 4, Table 4).

**Table 4:** Comparisons of jumping performance of *Rana temporaria* froglets (Gosner stage 45) from different populations. Tadpoles originated from Germany (GER) and Croatia (CRO) and developed under three temperature regimes (OT, T15, T20). We compared adjusted maximal jumping distance, MJI (MJI = max. jump/LLI, cm) of froglets from different populations using Welch two sample *t*‐test (*t*‐values, *df*,* P*‐values). Sample size was *n* = 29 for OT‐CRO, and *n* = 30 for all other treatments

	OT‐GER – OT‐CRO			T15‐GER – T15‐CRO			T20‐GER – T20‐CRO		
	*t*‐value	*df*	*P*‐value	*t*‐value	*df*	*P*‐value	*t*‐value	*df*	*P*‐value
E15	6.92	42.67	**<0.0001**	11.98	40.14	**<0.0001**	9.61	52.47	**<0.0001**
E20	5.25	53.04	**<0.0001**	9.18	54.56	**<0.0001**	6.52	57.94	**<0.0001**
E25	9.77	52.34	**<0.0001**	11.96	47.24	**<0.0001**	6.46	57.99	**<0.0001**

Statistically significant differences (*P*‐values <0.05) are shown in bold.

## Discussion

We detected differences in developmental time, morphometry, body condition, and jumping performance of metamorphs from different *Rana temporaria* populations and treatments. Froglets from Croatia (CRO) metamorphosed faster, but were of same size (or larger) and in better body condition than those from Germany (GER). Furthermore, they had longer hind limbs and performed better under all experimental temperatures, even after size correction. Froglets from GER developed at lower temperatures (OT, T15) reached their maximal jumping performance already under 20°C, and did not improve much (T15) or at all (OT) under 25°C, while CRO froglets constantly improved jumping performance with temperature increase.

### Developmental time

The duration of the anuran larval period is determined by several, partly interacting factors (Wassersug and Sperry 1977; Downie et al. 2004). When conditions during development become unfavorable, it might be beneficial to initiate metamorphosis. Desiccation is fatal for larval amphibians, this risk being higher in warmer environments (Dittrich et al. 2016). Temperatures and therewith desiccation risk of *R. temporaria* breeding sites often increase toward the end of the larval period in our study regions, especially in Croatia. Indeed, CRO tadpoles metamorphosed faster than GER, in all treatments. In the outdoor treatment, they experienced higher temperatures than GER (mean developmental temperatures – OT‐CRO: 14.1°C, OT‐GER: 12.4°C). As higher developmental temperatures accelerate ectotherms' developmental rates (Atkinson 1994), the faster development of OT‐CRO could simply be a consequence of higher temperatures. However, even when raised in equal constant temperatures, CRO tadpoles still developed faster. The most plausible interpretation is that CRO population adapted to faster development, to minimize higher temperatures and desiccation risk, both for tadpoles and for metamorphs. Metamorphs may have more possibilities to select microhabitats with suitable temperatures in the terrestrial habitat.

Similarly to this interpretation, studies on Swedish populations of *R. temporaria* with differing breeding pond persistency showed that tadpoles from time‐constrained temporary ponds, with high desiccation risk, developed faster, compared to tadpoles from more permanent ponds (Lind and Johansson 2007; Lind et al. 2008, 2010). Locally adapted developmental rates have been also shown for populations under other environmental constraints. For example, populations at higher latitudes or altitudes, constrained by lower environmental temperatures and shorter activity period, developed faster compared to the populations with higher temperatures and longer activity period at lower latitudes or altitudes (Merilä et al. 2000; Laugen et al. 2003b; Muir et al. 2014). This allowed them to accumulate sufficient reserves before hibernation and avoid freezing in the breeding sites.

### Morphometric traits and body condition

Morphometric traits and body condition of froglets from GER and CRO were significantly affected by developmental treatment. Froglets from outdoor treatments in GER and CRO were bigger, heavier, and in better body condition compared to constant temperature treatments. This was expected, as they were kept under almost natural condition, including temperature fluctuation (Niehaus et al. 2012) and additional food sources (e.g., available detritus and naturally growing algae; Altig et al. 2007). A general temperature‐size rule (TSR) for ectotherms states that higher temperatures increase developmental rates, at the cost of smaller size (Ray 1960; Atkinson 1994; Angilletta et al. 2004). In the CRO constant temperature treatments, our results were consistent with TSR. In contrast, GER‐T15 froglets were smaller than GER‐T20, without differences in mass or body condition. The reasons for this outcome are not entirely clear; however, a similar effect has previously been shown in some populations of this species (e.g., populations from central Sweden, Richter‐Boix et al. 2010; Scotland, Muir et al. 2014; or Pyrenees, Oromi et al. 2015).

We also detected significant differences in morphometric traits and body condition between froglets from different populations, reared under equal conditions. As outlined above, tadpoles often trade‐off timing and size at metamorphosis (Wilbur and Collins 1973; Rowe and Ludwig 1991). Interestingly, this was not the case in our study. Faster metamorphosing CRO froglets were of the same size (OT, T20) or bigger (T15), heavier, and in better body condition (without statistical support at T20), compared to GER. The possibility of faster development without body size reduction was likewise reported for *R. temporaria* populations facing environmental constraints in temporary ponds (Lind et al. 2008), or on latitudinal (Merilä et al. 2000) and altitudinal (Muir et al. 2014) scales.

What are the possible explanations of differences in morphometric traits between populations from Germany and Croatia? In warmer environments, bigger body size and/or mass can be an advantage for amphibians; for instance, it can lower desiccation risk (Thorson 1955; Atkinson 1994), as shown in mass‐specific evaporation rates in desert dwelling *Scaphiopus couchii* toadlets (Newman and Dunham 1994). Additionally, CRO froglets of all developmental treatments had longer hind limbs than GER. Higher body temperatures increase metabolic rates (Gillooly et al. 2001), which increases energy consumption and required food uptake (Atkinson 1994). Increased activity may lead to a higher risk of predation (Huey and Slatkin 1976). Therefore, attributes which improve locomotor performance (longer limbs and bigger body size; Zug 1972, 1978; this study) and thus improve foraging (Putnam and Bennett 1981) and reduce predation pressure (Wassersug and Sperry 1977) should be an advantage in warmer environments. Consequentially, better body condition and longer hind limbs could represent a local adaptation to higher energetic requirement of the CRO population.

A study of *R. temporaria* extremity length across Scandinavia (Alho et al. 2011) partially supports our findings, showing a trend of leg length decrease with latitude. However, this result stemmed from a common garden experiment, but was not observed under natural conditions. So far, no consistent large‐scale latitudinal or altitudinal trend of body size has been found in wild *R. temporaria* populations, despite size clines at smaller scales (Miaud et al. 1999; Laugen et al. 2005; Sinsch et al. 2015). It is probable that some of the experimentally observed patterns, such as geographically scaled growth and developmental rates or morphometric trends, are overridden by other environmental influences under natural conditions (Laugen et al. 2003b; Alho et al. 2011).

### Jumping performance

The overall influence of the developmental treatment on metamorphs' jumping performance was similar in both populations. In GER and CRO, froglets from outdoor treatments were the best jumpers (max. jump index, MJI). As outlined, outdoor treatment provided more favorable environment than constant temperature treatments, resulting in improved morphometric traits and body condition, as well as better jumping performance (Altig et al. 2007; Niehaus et al. 2012). T20‐froglets jumped longer distances (MJI) than T15, in both GER and CRO. Similar results were obtained for two other European anurans, *Pelophylax lessonae* (Orizaola and Laurila 2009) and *Discoglossus galganoi* (Álvarez and Nicieza 2002). Higher temperatures presumably enhance development of limb musculature *via* increased activity (Goldspink 1983) and influence on the muscle‐fiber number, size or structure (Stickland et al. 1988; Vieira and Johnston 1992).

Local adaptations of locomotion traits to variation in thermal environment have been previously reported for anurans, on longitudinal and altitudinal scales (John‐Alder et al. 1988; Navas 1996). For example, populations of the Australian frog *Limnodynastes peronii* from colder environments performed better at lower temperatures than those from warmer environment, and *vice versa* (Wilson 2001). We hypothesized thus that froglets from colder GER would perform better at lower temperatures, compared to those from warmer CRO. Contrary to our expectations, CRO froglets jumped further in all experimental trials, even after size correction. Again, it is possible that increased energy demands and predation risk of a warmer environment lead to enhanced jumping performance in CRO. Furthermore, the comparison of jumping performance thermal sensitivity revealed population‐specific reactions (Fig. 4). In CRO, MJI increased constantly with the experimental temperature (15°, 20° and 25°), in all treatments. In GER, however, influence of temperature differed among developmental treatments. Froglets developing at lower temperatures (OT and T15) reached their performance maximum when jumping under 20°C and did not improve much (T15), or at all (OT), with rising temperature to 25°C. Local adaptation to lower temperatures in the native habitat of GER could have led to the development of lower thermal optima for jumping performance, allowing maximal performance under lower temperatures than necessary for CRO. In a population from northern Poland, Köhler et al. (2011) detected maximal jumping performance already under 15°C, which is lower than in either GER or CRO. This supports the hypothesis that thermal sensitivity of jumping performance is subjected to local adaption. Metamorphs from T20‐GER, however, constantly improved performance with temperature increase, probably due to thermal acclimation. Previous studies have demonstrated that warm‐acclimated frogs and toads perform better when tested at higher temperatures than cold‐acclimated (Marsh 1994).

## Conclusions

Even when reared in common environments (constant temperature treatments), *R. temporaria* metamorphs from two geographically distant populations differed in developmental time, morphometrics, and jumping performance. Common environments remove environmental effects, others than the one(s) tested for, hence indicating genetics as the source, in a case of observed variation. Such differences might be especially important in the light of ongoing (and predicted) environmental change. Organisms may survive environmental changes by migrating to suitable areas, phenotypic plasticity or genetic adaptation. If they cannot apply any of these responses, populations, or even species, face extinction (Urban et al. 2014). Migration to more favorable habitats is often limited by the accessibility of new areas (Schloss et al. 2012), and/or low dispersal capacity (Sinsch 1990). Therefore, besides the benefits of increased performance when physiologically adapted to the respective thermal conditions, adaptive potential might be a crucial response enabling survival in a changing environment. On the other hand, locally adapted populations might be more subjected to negative effects of the change. Thus, to assess the impact of potential variation in thermal conditions, it is essential to determine population‐specific potential of thermal adaptation.

## Data accessibility

All data used are presented in the manuscript and the Appendix to the manuscript.

## Conflict of Interest

None declared.

## Supporting information


**Figure S1.** Daily temperature fluctuations in the outdoor treatment.
**Table S1.** Relationship among morphometric traits, body condition and leg length index, and jumping performance of *Rana temporaria* froglets (Gosner stage 45).
**Table S2.** Overall influence of developmental treatment and experimental temperatures on jumping performance of *Rana temporaria* froglets (Gosner stage 45).
