# Supplementary material for: Population‐specific effects of developmental temperature on body condition and jumping performance of a widespread European frog
Source: Ecol Evol. 2016 Apr 3;6(10):3115–28. doi: 10.1002/ece3.2113 (PMC4823144; doi:10.1002/ece3.2113)
Supplement: Supplementary file 1 — Figure S1. Daily temperature fluctuations in the outdoor treatment. Table S1. Relationship among morphometric traits, body condition and leg length index, and jumping performance of Rana temporaria froglets (Gosner stage 45). Table S2. Overall influence of developmental treatment and experimental temperatures on jumping performance of Rana temporaria froglets (Gosner stage 45). [file ECE3-6-3115-s001.docx]

**Appendix**


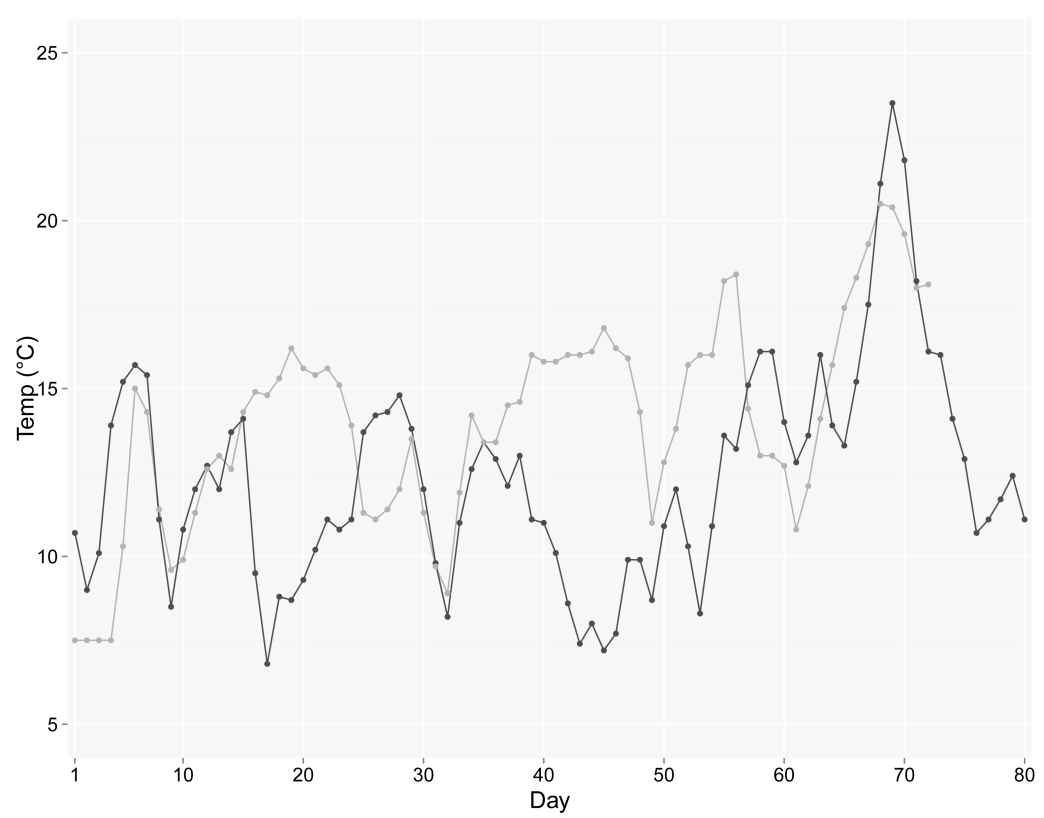


**Fig. A1. Daily temperature fluctuations in the outdoor treatment.** Mean daily temperatures (°C) in outdoor treatment during developmental period of *Rana temporaria* tadpoles (from the eggs collection, until metamorphosis of the last metamorph, n days: GER= 80, CRO= 72), from two different populations (Germany: GER, dark grey and Croatia: CRO, light grey). Temperatures are expressed as daily means of water temperatures in outdoor treatment experimental containers, recorded every three hours, across 10 iButton data loggers per location. Mean daily temperature of developmental period (n(GER)= 80, n(CRO)= 72) was significantly higher in CRO (mean= 14.0°C, range: 7.5-20.5°C, s.d.= 3.1) compared to GER (mean= 12.4°C, range: 6.8-23.5°C, s.d.= 3.2; Kruskal-Wallis test, χ^2^=13.55, *df*= 1, p= 0.0002).

**Table A1. Relationship among morphometric traits, body condition and leg length index, and jumping performance of *Rana temporaria* froglets (Gosner stage 45).** We analysed the correlations of morphometric traits (leg length (LL, mm), leg length index (LLI), mass (g), scaled mass index (SMI, g), and size (SVL, mm), and maximal jumping distance (max. jump, cm)). We pooled data for all treatments (outdoor treatment: OT, constant temperatures - 15°: T15 and 20°C: T20), and both population origins (Germany: GER and Croatia: CRO), but divided them by experimental temperatures of 15°C (E15), 20°C (E25) and 25°C (E25). LLI showed the highest correlation with max. jumping distance. Therefore, we adjusted maximal jumping distance for LLI, by calculating the max. jump index; MJI= max. jumping distance/LLI. Given is Pearson’s product-moment correlation coefficient for the correlation matrix. Sample size was n= 179 per experimental temperature.

| **Exp. temp.** | **LL** | **LLI** | **mass** | **SMI** | **SVL** |
| --- | --- | --- | --- | --- | --- |
| **E15** | 0.77 | **0.79** | 0.52 | 0.40 | 0.47 |
| **E20** | 0.78 | 0.74 | 0.59 | 0.44 | 0.54 |
| **E25** | 0.75 | **0.76** | 0.53 | 0.44 | 0.46 |

**Table A2. Overall influence of developmental treatment and experimental temperatures on jumping performance of *Rana temporaria* froglets (Gosner stage 45).** Tadpoles originated from A. Germany (GER) and B. Croatia (CRO), and developed under three temperature regimes (OT, T15, T20). We analysed the overall influence of developmental treatments and environmental temperatures on jumping performance of *Rana temporaria* froglets (Gosner stage 45) using linear mixed-effect models for max. jump index (MJI) with developmental treatment (Treatment) and experimental temperature (Experimental temperature) as fixed, and clutch as a random effect; for different populations separately. Sample size was n= 29 for OT-CRO, and n= 30 for all other treatments. Analysis was conducted using *lme* function from *nlme* package for R (Pinheiro et al. 2014, R Development Core Team 2014).

|  | **A. GER** (AIC= 950.13, *df*= 256) | | | **B. CRO** (AIC= 1086.18, *df*= 253) | | |
| --- | --- | --- | --- | --- | --- | --- |
| **Fixed effects** | ***estimate*** | ***t-value*** | ***p-value*** | ***estimate*** | ***t-value*** | ***p-value*** |
| **Intercept** | 8.97 | 42.77 | **<0.0001** | 11.74 | 40.54 | **<0.0001** |
| ***Treatment*** |  | | |  | | |
| T15 | -3.34 | -16.59 | **<0.0001** | -1.45 | -5.46 | **<0.0001** |
| T20 | -1.21 | -5.93 | **<0.0001** | -0.72 | -2.73 | **0.007** |
| ***Experimental temperature*** |  | | |  | | |
| E20 | 2.21 | 11.04 | **<0.0001** | 1.38 | 5.23 | **<0.0001** |
| E25 | 2.71 | 13.55 | **<0.0001** | 3.51 | 13.29 | **<0.0001** |
